# Supplementary material for: Sex-Specific Expression of Histone Lysine Demethylases (KDMs) in Thyroid Cancer
Source: Cancers (Basel). 2024 Mar 23;16(7):1260. doi: 10.3390/cancers16071260 (PMC11010840; doi:10.3390/cancers16071260)
Supplement: Supplementary file 1 [file cancers-16-01260-s001.zip › cancers-2923192-supplementary.pdf]

Supplemental Table S1

#	Symbol	Gene name	Upregulated in male (M) or female (F)	Linked with X/Y chromosomes
1	ABHD10	abhydrolase domain containing 10	M	NO
2	ACRBP	acrosin binding protein	M	NO
3	ACTR3C	ARP3 actin related protein 3 homolog C	M	NO
4	AGPAT3	1-acylglycerol-3-phosphate O-acyltransferase 3	M	NO
5	AMELY	amelogenin, Y-linked	M	YES
6	APITD1	centromere protein S	M	NO
7	BCORL2	Also known as BCORP1 BCL6 corepressor pseudogene 1	M	YES
8	C17orf97	chromosome 17 open reading frame 97	M	NO
9	CRISP2	cysteine rich secretory protein 2	M	NO
10	CWH43	cell wall biogenesis 43 C-terminal homolog	M	NO
11	CYorf15A	TXLNGY taxilin gamma pseudogene, Y-linked	M	YES
12	CYorf15B	TXLNGY taxilin gamma pseudogene, Y-linked	M	NO
13	DDX3Y	DEAD-box helicase 3, Y-linked	M	YES
14	EIF1AY	eukaryotic translation initiation factor 1A, Y-linked	M	YES
15	EPHB2	EPH receptor B2	M	NO
16	FAM125B	MVB12B multivesicular body subunit 12B	M	NO
17	HSFY2	heat shock transcription factor, Y-linked 2	M	YES
18	KDM5D	lysine demethylase 5D	M	YES
19	MAPK9	mitogen-activated protein kinase 9	M	NO
20	NCRNA001	TTTY14 testis-specific transcript, Y-linked 14 (non-protein coding)	M	YES
21	NCRNA002	FAM224B family with sequence similarity 224 member B (non-protein coding)	M	YES
22	NLGN4Y	neuroligin 4, Y-linked	M	YES
23	PATZ1	POZ/BTB and AT hook containing zinc finger 1	M	NO
24	PCDH11Y	protocadherin 11 Y-linked	M	YES
25	PRKY	protein kinase, Y-linked, pseudogene	M	YES
26	PTCD2	pentatricopeptide repeat domain 2	M	NO
27	RBM46	RNA binding motif protein 46	M	NO
28	RPS4Y1	ribosomal protein S4, Y-linked 1	M	YES
29	RPS4Y2	ribosomal protein S4, Y-linked 2	M	YES
30	SEPHS1	selenophosphate synthetase 1	M	NO
31	ST6GAL2	ST6 beta-galactoside alpha-2,6-sialyltransferase 2	M	NO
32	TBL1Y	transducin beta like 1 Y-linked	M	YES
33	TMSB4Y	thymosin beta 4	M	YES
34	TRAM1L1	translocation associated membrane protein 1-like 1	M	NO
35	TTTY10	testis-specific transcript, Y-linked 10	M	YES
36	TTTY14	testis-specific transcript, Y-linked 14	M	YES
37	TTTY15	testis-specific transcript, Y-linked 15	M	YES
38	TXNDC15	thioredoxin domain containing 15	M	NO
39	USP9Y	ubiquitin specific peptidase 9, Y-linked	M	YES
40	UTY	ubiquitously transcribed tetratricopeptide repeat containing, Y-linked	M	YES
41	ZBED1	zinc finger BED-type containing 1	M	YES
42	ZFY	zinc finger protein, Y-linked	M	YES
43	ZNF396	zinc finger protein 396	M	NO
44	ZNF709	zinc finger protein 709	M	NO
45	CA5BP	carbonic anhydrase 5B pseudogene 1	F	YES
46	DDX3X	DEAD-box helicase 3, X-linked	F	YES
47	EIF1AX	eukaryotic translation initiation factor 1A, X-linked	F	YES
48	EIF2S3	eukaryotic translation initiation factor 2 subunit gamma	F	YES
49	HDHD1A	pseudouridine 5'-phosphatase	F	YES
50	IL12A	interleukin 12A	F	NO
51	INPP1	inositol polyphosphate-1-phosphatase	F	NO
52	ITGA5	integrin subunit alpha 5	F	NO
53	KDM5C	lysine demethylase 5C	F	YES
54	KDM6A	lysine demethylase 6A	F	YES
55	LCN6	lipocalin 6	F	NO

56	MYO1A	myosin IA	F	NO
57	NCRNA00:	JPX transcript, XIST activator (non-protein coding)	F	YES
58	OFD1	centriole and centriolar satellite protein	F	YES
59	PIM1	Pim-1 proto-oncogene, serine/threonine kinase	F	NO
60	PIP5K1B	phosphatidylinositol-4-phosphate 5-kinase type 1 beta	F	NO
61	PNPLA4	patatin like phospholipase domain containing 4	F	YES
62	PPP1R2P9	protein phosphatase 1 regulatory inhibitor subunit 2 pseudogene 9	F	YES
63	RAET1L	retinoic acid early transcript 1L	F	NO
64	RPS4X	ribosomal protein S4, X-linked	F	YES
65	SPAG6	sperm associated antigen 6p	F	NO
66	SYAP1	synapse associated protein 1	F	YES
67	TAGLN2	transgelin 2	F	NO
68	TSIX	TSIX transcript, XIST antisense RNAp	F	YES
69	VAX2	ventral anterior homeobox 2	F	NO
70	XIST	X inactive specific transcript	F	YES
71	ZRSR2	zinc finger CCCH-type, RNA binding motif and serine/arginine rich 2	F	YES
72	ZYX	zyxin	F	NO

Supplemental Table S2

Symbol	Entrez Gene Name	Location	Family	Gene ID
COPG2	COPI coat complex subunit gamma 2	Cytoplasm	transporter	26958
INPP1	inositol polyphosphate-1-phosphatase	Cytoplasm	phosphatase	3628
ITGA5	integrin subunit alpha 5	Plasma Membrane	transmembrane receptor	3678
LCN6	lipocalin 6	Extracellular Space	other	158062
MYO1A	myosin IA	Cytoplasm	peptidase	4640
PUDP	pseudouridine 5'-phosphatase	Cytoplasm	enzyme	8226
RAET1L	retinoic acid early transcript 1L	Plasma Membrane	other	154064
RPS4X	ribosomal protein S4 X-linked	Cytoplasm	other	6191
TAGLN2	transgelin 2	Cytoplasm	other	8407
VAX2	ventral anterior homeobox 2	Nucleus	transcription regulator	25806
ZRSR2	zinc finger CCCH-type, RNA binding motif and serine/arginine rich 2	Nucleus	other	8233
ZYX	zyxin	Plasma Membrane	other	7791

Supplemental Table S3

Symbol	Entrez Gene Name	Location	Family	Gene ID
ABHD10	abhydrolase domain containing 10, depalmitoylase	Cytoplasm	enzyme	55347
ACRBP	acrosin binding protein	Extracellular Space	other	84519
ACTR3C	actin related protein 3C	Cytoplasm	other	653857
AGPAT3	1-acylglycerol-3-phosphate O-acyltransferase 3	Cytoplasm	enzyme	56894
AMELY	amelogenin Y-linked	Extracellular Space	growth factor	266
CWH43	cell wall biogenesis 43 C-terminal homolog	Other	other	80157
DDX3X	DEAD-box helicase 3 X-linked	Cytoplasm	enzyme	1654
EIF1AX	eukaryotic translation initiation factor 1A X-linked	Cytoplasm	translation regulator	1964
EPHB2	EPH receptor B2	Plasma Membrane	kinase	2048
IL12A	interleukin 12A	Extracellular Space	cytokine	3592
KDM6A	lysine demethylase 6A	Nucleus	enzyme	7403
PCDH11Y	protocadherin 11 Y-linked	Other	other	83259
PIM1	Pim-1 proto-oncogene, serine/threonine kinase	Cytoplasm	kinase	5292
PNPLA4	patatin like phospholipase domain containing 4	Cytoplasm	enzyme	8228
PPP1R2C	PPP1R2C family member C	Other	other	80316
PRKY	protein kinase Y-linked (pseudogene)	Other	other	5616
PTCD2	pentatricopeptide repeat domain 2	Cytoplasm	other	79810
RBM46	RNA binding motif protein 46	Nucleus	other	166863
SEPHS1	selenophosphate synthetase 1	Other	enzyme	22929
SEPHS1P:	selenophosphate synthetase 1 pseudogene 1	Other	other	168474
SYAP1	synapse associated protein 1	Nucleus	other	94056
TRAM1L1	translocation associated membrane protein 1 like 1	Extracellular Space	other	133022
TXNDC15	thioredoxin domain containing 15	Extracellular Space	enzyme	79770
ZNF396	zinc finger protein 396	Nucleus	transcription regulatoı	252884
ZNF709	zinc finger protein 709	Other	other	163051
